# Taiwanese Green Propolis Ethanol Extract Delays the Progression of Type 2 Diabetes Mellitus in Rats Treated with Streptozotocin/High-Fat Diet

**DOI:** 10.3390/nu10040503

**Published:** 2018-04-18

**Authors:** Li-Han Chen, Yi-Wen Chien, Mei-Ling Chang, Chia-Chung Hou, Ching-Hung Chan, Hung-Wei Tang, Hui-Yu Huang

**Affiliations:** 1 Graduate Institute of Biomedical Electronics and Bioinformatics, National Taiwan University, Taipei City 10617, Taiwan; lihan.h.chen@gmail.com; 2 School of Nutrition and Health Sciences, Taipei Medical University, Taipei City 11031, Taiwan; ychien@tmu.edu.tw; 3 Research Center of Geriatric Nutrition, College of Nutrition, Taipei Medical University, Taipei City 11031, Taiwan; 4 Graduate Institute of Metabolism and Obesity Science, Taipei Medical University, Taipei City 11031, Taiwan; 5 Department of Food Science, Nutrition, and Nutraceutical Biotechnology, Shih Chien University, Taipei City 10462, Taiwan; mlchang@g2.usc.edu.tw (M.-L.C.); llfonly_520@hotmail.com (C.-H.C.); 9808408@gmail.com (H.-W.T.); 6 Department of Research & Development, NatureWise Biotech & Medicals Corporation, Taipei City 10559, Taiwan; alison.hou@naturewise.com.tw

**Keywords:** Taiwanese green propolis extract, type 2 diabetes mellitus, anti-oxidation, anti-inflammation, *PPAR-α*, *CYP7A1*

## Abstract

Taiwanese green propolis ethanol extract (TGPE) is produced only in Taiwan and has a different composition from other types of propolis. TGPE is known for its anti-inflammation, anti-oxidation, and anti-microbial properties, but the effects and mechanisms of TGPE in the modulation of diabetes are unclear. In this study, we investigated the effects of TGPE on type 2 diabetes mellitus (T2DM) in a streptozotocin/high-fat-diet (STZ/HFD)-induced T2DM rat model. The results revealed that TGPE delayed the development and progression of T2DM and reduced the severity of β-cell failure. TGPE also attenuated inflammation and reactive oxygen species ROS in the rats. Moreover, there were higher levels of oxidant cytokines, leptin, and adiponectin in the serum of the TGPE-treated group. Unlike Brazilian propolis, TGPE promoted hepatic genes *PPAR-α* and *CYP7A1*, which were related to lipid catabolism and removal. TGPE may thus delay the progression of T2DM through anti-inflammation effects, anti-oxidation effects, and balancing lipid metabolism. It is suggested that TGPE can be a potential alternative medicine for T2DM.

## 1. Introduction

Diabetes mellitus is a common endocrine disease that leads to many dangerous complications. In 2015, there were 415 million people who suffered from diabetes worldwide, and this number is estimated to rise to 642 million by 2040 [1]. The direct annual cost of treating diabetes has reached $825 billion since 2014 [2]. Thus, many researchers are seeking to develop alternative medicinal foods to improve diabetes [3].

Propolis is a safe, natural product that contains bee secretions derived from various plant sources. Propolis has been used as a folk medicine in many Eastern and Western countries because of its anticancer [4], antioxidant [4], anti-microbial [5,6], and anti-inflammatory [7] properties. Brazilian propolis has been reported to have effects on hyperglycemia and hyperlipidemia [8]. However, the mechanisms of these effects are still unclear. Furthermore, the compositions of green propolis vary from ecosystem to ecosystem due to the differences in local flora.

Taiwanese green propolis extract (TGPE) has been widely used in traditional medicine in Taiwan. Su et al. (2014) demonstrated that TGPE contains propolins C, D, F, G, and H, which can influence several hepatic genes and protect the liver from fibrosis [9]. Moreover, TGPE has been shown to have antioxidant and antimicrobial properties [10,11]. However, no studies have explored the effects of TGPE on the progression of diabetes.

The development of type 2 diabetes mellitus (T2DM) involves a reduction in insulin sensitivity with high basal insulin secretion and hyperproinsulinemia [12]. The pathological conditions arise in the early stages of the disease progression of T2DM and before the β cells severely fail in the late stage [13]. Therefore, the progression of T2DM can be delayed by improving the sensitivity of insulin receptors, especially in those of hepatic cells, muscle cells, and adipocytes. The decline in β-cell function is another factor that causes the advance of T2DM. Thus, maintaining the mass and function of β-cells is also important for alleviating the course of T2DM.

Increases in reactive oxidant species (ROS) and inflammation lead to insulin resistance and β-cell failure. Therefore, enhancing the body’s antioxidant and anti-inflammatory capabilities could be a strategy to slow down the development and progression of T2DM. Moreover, because T2DM is also often associated with obesity [14], another possible solution is to focus on the downregulation of fat accumulation. This could be achieved by decreasing dietary fat absorption via gastrointestinal bypass or by increasing lipid catabolism via fat removal. Hepatic peroxisome proliferator-activated receptor-α (*PPAR-α*) is known to increase lipid catabolism and decrease lipid storage. Hepatic cholesterol-7a-hydroxylase (*CYP7A1*) is a rate-limiting enzyme involved in the de novo synthesis of bile acid [15] and can maintain the homeostasis of cholesterol metabolism [16,17].

TGPE has been reported to attenuate oxidation, reduce inflammation, and regulate several hepatic genes in the liver. Therefore, we hypothesized that TGPE supplements could help modulate the development and progression of T2DM. We also investigated the potential molecular mechanism of TGPE in delaying T2DM.

## 2. Materials and Methods

### 2.1. Sample Preparation and HPLC Analyses

TGPE was provided by NatureWise Biotech & Medicals Corporation (Taipei, Taiwan). All TGPE was collected from May to July and extracted with 95% ethanol (250 mL × 3), sonicated for 3 h, and left to stand at 25 °C for 21 h. The extract was evaporated to dryness and kept at −20 °C until processed. HPLC was performed to analyse the TGPE with the following conditions: column: Phenomenex Luna 3-μm C18(2) 250 × 4.6 mm; mobile phase: gradient methanol/water; flow rate: 1.0 mL/min; temperature: 25 °C; detection: UV 280 nm; injection volume: 20 μL.

The recommended dose of TGPE for humans is 1779.51 mg/60 kg/day according to NatureWise. According to a report from the United States Food and Drug Administration (FDA), the conversion constant between rats and humans is 6.2 [18]. Therefore, the recommended dose for rats is 29.66 mg/kg/day × 6.2 = 183.9 mg/kg/day. Since the conversion constant is 1.98 between rats and mice, the dose is equivalent to 364.12 mg/kg/day in mice, which is in a range that increased GPx and SOD in mice [9].

### 2.2. Animal Model

A total of 40 male Sprague Dawley (SD) rats (eight weeks old, ~270 g) were purchased from a local supplier (LASCO, Taipei, Taiwan). All rats were housed individually under standard laboratory conditions (12/12 h light/dark cycle, 22–24 °C, 40–60% humidity) with free access to food and water. The control group (C) comprised 10 rats given a normal diet (see supplemental Table S1 for the ingredient composition) with distilled water and were intraperitoneally injected with phosphor-buffered saline (PBS) every two days until week 4. The other 30 rats were randomly selected and assigned to three groups: the DM group (diabetes control, given distilled water), the 1X group (given 183.9 mg/kg/day of TGPE), and the 5X group (given 919.5 mg/kg/day of TGPE). These three groups were fed with a high-fat and -fructose diet (HFD, see supplemental Table S1 for ingredient composition) and intraperitoneally injected with streptozotocin (STZ) at 15 mg/kg every two days from week 1 to week 4. The animal model was modified from our previous study [19], which used multiple low doses of STZ and HFD to induce T2DM.

The powdered TGPE was dissolved in 1 mL of distilled water and administered by gavage to the 1X and the 5X groups at 9 a.m. every day for 8 weeks. In contrast, the C and DM groups received 1 mL of distilled water instead. Food and water intakes were recorded every day, and body weights (BWs) were measured weekly. The rats were sacrificed at the eighth week. All animal experiments were performed in accordance with the protocols approved by the Institutional Animal Care and Use Committee (IACUC) of Shih Chien University (IACUC-10305).

### 2.3. Serum Biochemical Analysis

After the rats had fasted for 8 h, blood was collected to determine their fasting blood glucose (FBG) using the Accu-Chek system (Roche, Indianapolis, IN, USA) and centrifuged at 3500× *g* for 10 min at 4 °C to obtain serum. Fasting blood insulin (FBI) was detected using a Mercodia Rat Insulin ELISA Kit (Mercodia, Uppsala, Sweden). Serum glycated hemoglobin (HbA1c), triacylglycerol (TG), total cholesterol (TC), low-density lipoprotein (LDL), and high-density lipoprotein (HDL) levels were measured using SYNCHRON^®^ Systems (Beckman Coulter Inc., Fullerton, CA, USA). HOMA-IR was calculated according to Park et al. (2009) [20] as HOMA-IR = insulin (μU/mL) × glucose (mmol/L)/22.5.

### 2.4. Insulin Sensitivity Indices (ISI)

ISI was calculated according to FBG and FBI as follows:

ISI = Ln (FBG × FBI) − 1

### 2.5. Oral Glucose Tolerance Test (OGTT)

Oral glucose tolerance tests were performed at the eighth week. The rats were removed from their cages for 8 h before the beginning of the tests. After giving an oral glucose load (1 g/kg of body weight) by oral gavage, blood samples were collected from the tail vein at 0, 30, 60, 90, 120, and 180 min. Glucose and insulin concentrations were determined using the Accu-Chek system (Roche) and SYNCHRON^®^ Systems (Beckman Coulter Inc.), respectively. The total glucose and insulin areas under the curve (AUCglucose and AUCinsluin) between 0 and 180 min were used to represent the magnitude of the glucose and insulin responses and were calculated as described previously [21].

### 2.6. β-Cell Function and Mass

β-cell function (HOMA-β) was calculated according to Park et al. (2009) [20] as HOMA-β = (20 × fasting insulin)/(fasting glucose-3.5). The pancreases were weighed after sampling and then fixed in 4% paraformaldehyde solution, embedded in paraffin blocks, and sliced into 4 μm sections. Sections were immunostained with guinea pig anti-insulin IgG (Millipore, Billerica, MA, USA) for 1 h, followed by horseradish peroxidase-conjugated secondary antibody (Millipore) for 1 h. The sections were then counterstained with hematoxylin and digitally imaged (3DHISTECH, Budapest, Hungary).

### 2.7. Enzyme-Linked Immunosorbent Assay

Serum pro-inflammatory factors were determined using a sandwich ELISA kit for tumour necrosis factor-α (TNF-α) (Biolegend, San Diego, CA, USA), interlukin-6 (IL-6) (Peprotech, Rocky Hill, NJ, USA), and interlukin-1β (IL-1β) (Peprotech). Serum levels of leptin and adiponectin were measured using a leptin and adiponectin ELISA kit (Millipore). All assays were performed according to the instructions from the manufacturer. Serum samples were run undiluted. Optical density (OD) values were detected using an ELISA reader.

### 2.8. Antioxidants Activity and Oxidative Stress Levels

Superoxide dismutase (SOD) and glutathione peroxidase (GPx) activities were measured in serum using SOD and GPx assay kits (Cayman Chemicals Inc., Ann Arbor, MI, USA) according to the manufacturer’s instructions. The SOD and Gpx activities of the samples were calculated using an equation obtained from the linear regression of the standard curve. Thiobarbituric acid reactive substances (TBARS) were also analyzed using a TBARS assay kit (Cayman Chemical) to evaluate the oxidative stress in serum.

### 2.9. RNA Extraction and Quantitative RT-PCR

RNA was isolated from the rats’ livers using an RNeasy Mini kit (Qiagen, Hilden, Germany), and 500 ng of RNA from each sample was reverse-transcribed using an iScript cDNA synthesis kit (Bio-Rad, Hercules, CA, USA) according to the manufacturer’s instructions. Quantitative PCR was performed using SYBR green master mix (BioRad) in a MyiQ Single-Color Real-Time PCR Detection System (Bio-Rad). The sequences of the gene-specific primers (Purigo, Taipei, Taiwan) of *PPAR-α*, *CYP7A1*, sterol regulatory element-binding protein (SREBP), and *β-Actin* are shown in supplemental Table S2. *β-Actin* was used as an internal control for normalizing the mRNA levels of tested genes.

### 2.10. Statistical Analyses

To evaluate the progression of T2DM in the DM group (diabetes control), a one-tailed student’s *t*-test was performed to analyze the difference between the C and DM groups. To investigate the effects of TGPE on the progression of T2DM, one-way ANOVA with a Duncan post hoc test was used to analyze the data of the DM, 1X, and 5X groups. The results are presented as the means ± standard error of the mean (SEM). A *p* value < 0.05 was considered statistically significant.

## 3. Results

### 3.1. Chemical Composition of TGPE

**Figure 1:**
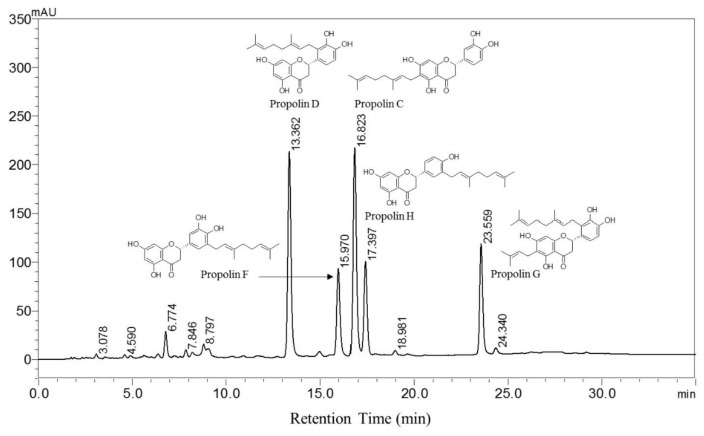
The HPLC chromatogram of Taiwan green propolis extracts. The conditions were as follows: column: Phenomenex Luna 3 μm C18(2) 250 × 4.6 mm; mobile phase: gradient methanol/water; flow rate: 1.0 mL/min; temperature: 25 °C; detection: UV 280 nm; injection volume: 20 μL. Peaks: 1: propolin D; 2: propolin F; 3: propolin C; 4: propolin H; 5: propolin G.

HPLC analyses of the TGPE identified five major compounds: propolins C, D, F, G, and H. Propolin C is a main component with the highest content, as shown in Figure 1.

### 3.2. BW Gain, Feed Conversion Efficiency (FCE), and Water Intake

BW increased during the experiment in all groups (supplementary Figure S1). The BW gains were between 196.6 and 174.0 g and significantly lower in the DM group than in the other groups. Although the total calorie intake was higher in the 1X and 5X groups than in the C group, the BW gains of these groups were only slightly different from those of the C group (Table 1). The DM group had the lowest FCE among the four groups, followed by the 1X and 5X groups, with the C group showing the highest FCE (Table 1).

Increased water intake is another symptom of diabetes. There were no initial differences in water intake between the groups, but by the eighth week, the DM group had much higher water intake than the others. The groups in order of lowest to highest water intake are C, 5X, 1X, and DM at the eighth week (Table 1).

### 3.3. Blood Glucose, Insulin, ISI, and OGTT

The concentrations of FBG and FBI were analyzed to assess the effects of TGPE on delaying STZ/HFD-induced diabetes. In the eight-week period, the concentration of FBG remained stable in the C group. However, it increased from 86.5 ± 6.7 to 392.4 ± 39.0 mg/dL in the DM group, from 85.8 ± 4.2 to 218.7 ± 38.4 mg/dL in the 1X group, and from 86.0 ± 7.1 to 141.8 ± 9.8 mg/dL in the 5X group.

**Figure 2:**
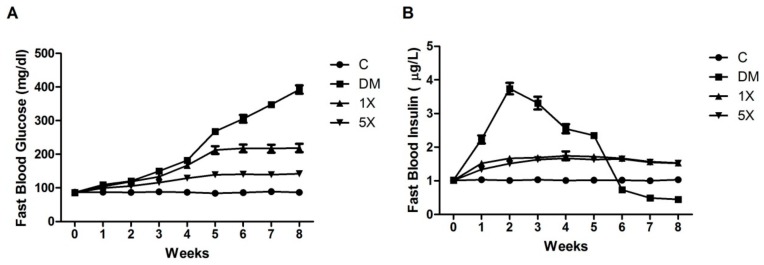
Effects of TGPE on FBG and FBI. The levels of FBS (**A**) and FBI (**B**) over eight weeks. C: vehicle control; DM: STZ/HFD vehicle control; 1X: STZ/HFD with 183.9 mg/kg/day of TGPE; 5X: STZ/HFD with 919.5 mg/kg/day of TGPE. Data are the means ± SEM (*n* = 10 rats/group).

The FBG curve of the DM group quickly increased to 181.8 ± 8.1 mg/dL at week 4 when STZ injections were stopped and then rose steeply until the end. However, the curves of the TGPE-treated groups increased only slightly (Figure 2A). The concentrations of FBI remained steady in the C group, while there was an increase of approximately 50% in the 1X and 5X groups. In the DM group, the concentrations of FBI reached 3.74 ± 0.53 μg/L at week 2, suddenly decreased at week 6, and was 0.45 ± 0.16 μg/L by week 8 (Figure 2B). ISI and HOMA-IR can be used to evaluate insulin sensitivity and insulin resistance, respectively, but they are only accurate before β-cell failure. Therefore, we compared ISI and HOMA-IR between groups at the fifth week. ISI was highest in the DM group, followed by the 1X, 5X, and C groups. HOMA-IR was highest in the C group, followed by the 5X, 1X, and DM groups (Table 2). Thus, TGPE improved insulin sensitivity and insulin resistance in STZ/HFD treated rats.

**Figure 3:**
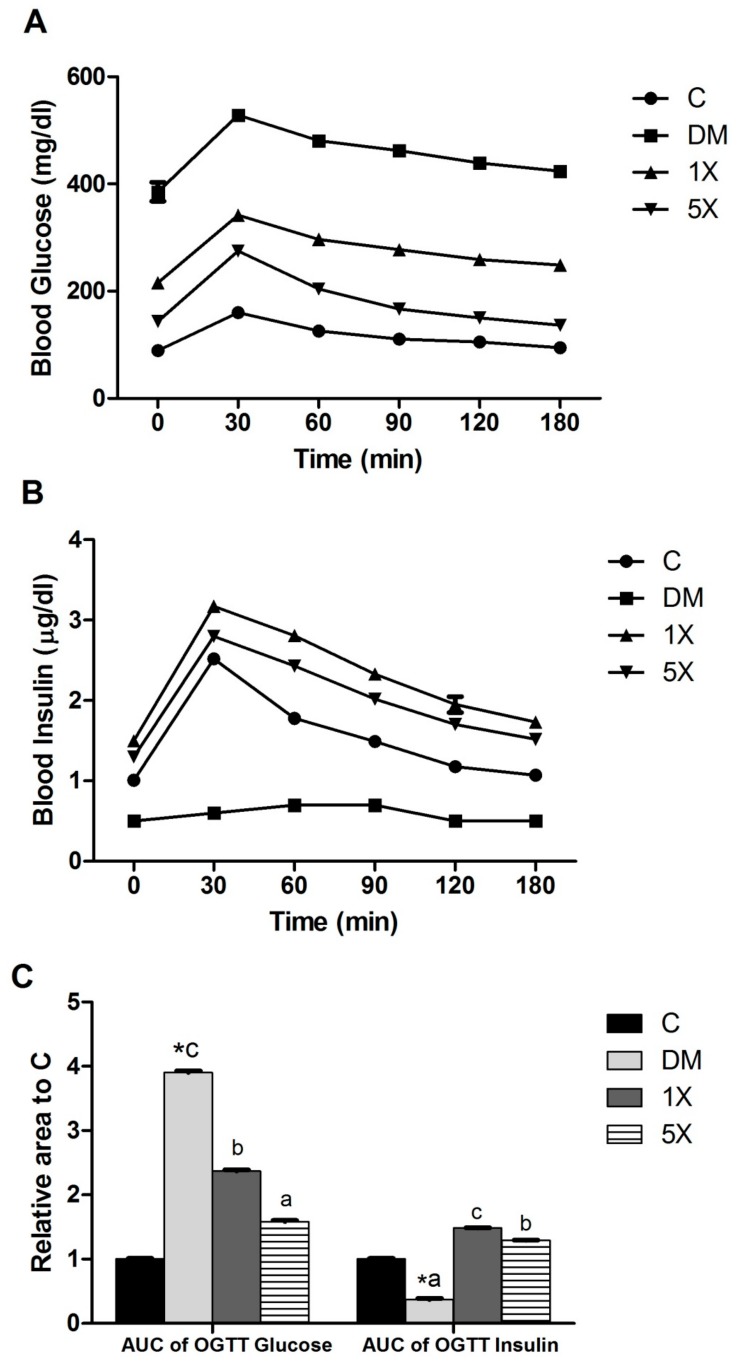
Glucose tolerance test. (**A**) Glucose; (**B**) insulin; and (**C**) area under the curve (AUC) at the eighth week. C: vehicle control; DM: STZ/HFD vehicle control; 1X: STZ/HFD with 183.9 mg/kg/day of TGPE; 5X: STZ/HFD with 919.5 mg/kg/day of TGPE. Data are the mean ± SEM (*n* = 10 rats/group). * Significantly different from the C group at *p* < 0.05 according to a one-tailed student’s *t*-test. Different superscript letters (a, b, c) indicate a significant difference at *p* < 0.05 according to one-way ANOVA with a Duncan post hoc test.

By the end of week 8, the DM group had the highest concentrations of FBG and HbA1c, followed by the 1X, 5X, and C groups (Table 2). The results of the OGTT assay showed that the levels of blood glucose and insulin increased between 0 and 30 min and decreased afterward in all groups. The DM group had the highest blood glucose levels and the lowest insulin during the whole period. Moreover, higher OGTT_glucoseAUC_ and lower OGTT_insulinAUC_ were observed in the DM group than in the C group (Figure 3).

The results indicate that STZ/HFD successfully induced T2DM because the treatment led to hypoglycemia and other important symptoms of T2DM, glucose intolerance, insulin resistance, and insulin release disorder. Furthermore, the TGPE treated groups had lower OGTT_glucoseAUC_ and higher OGTT_insulinAUC_ compared to the DM group. Therefore, the STZ/HFD-induced disorders of blood glucose, insulin, and insulin resistance were modulated in the TGPE-treated groups, especially the 5X group.

### 3.4. β-Cell Function and Mass

**Figure 4:**
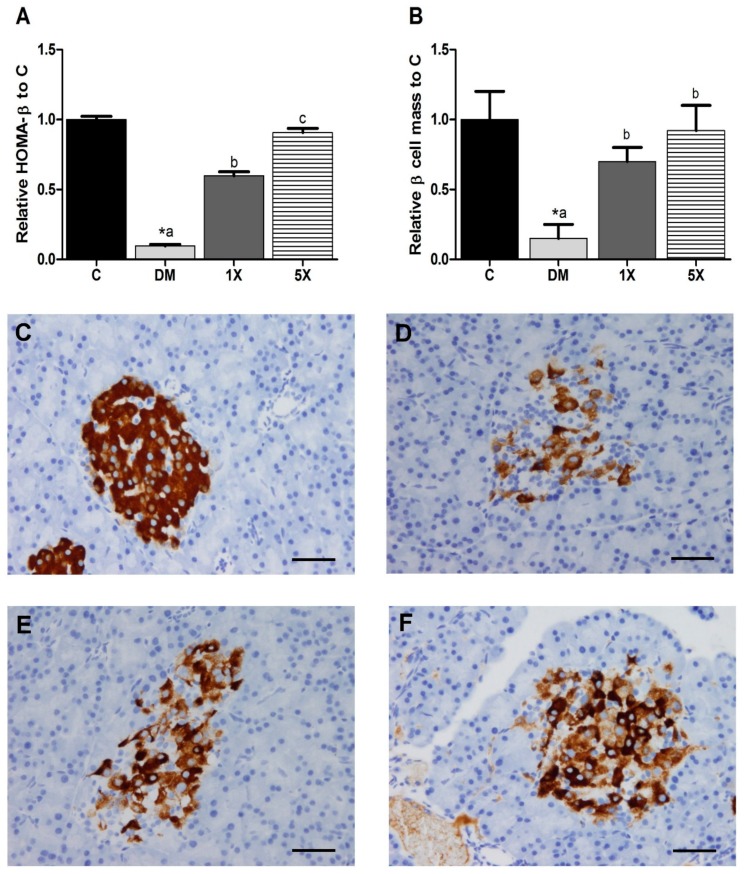
β-cell function and immunohistochemisty analysis of β-cells stained with pig anti-insulin IgG and hematoxylin. The β-cell function is displayed by the relative HOMA-β (**A**); β-cell mass (**B**) was calculated by multiplying the β cell/islet cell × the islet area/pancreas area × the pancreas weight. Sections of β-cells of the C (**C**); DM (**D**); 1X (**E**); and 5X (**F**) groups are noted. C: vehicle control; DM: STZ/HFD vehicle control; 1X: STZ/HFD with 183.9 mg/kg/day of TGPE; 5X, STZ/HFD with 919.5 mg/kg/day of TGPE. The scale bars are the black bars at the bottom-right corners of (**C**–**F**). Data are the means ± SEM (*n* = 10 rats/group). * Significantly different from the C group at *p* < 0.05 according to a one-tailed student’s *t*-test. Different superscript letters (a, b, c) indicate a significant difference at *p* < 0.05 according to one-way ANOVA with a Duncan post hoc test. Scare bar = 50 μm.

Since TGPE demonstrated a significant impact on FBG and FBI, we further investigated the condition of insulin secreting β-cells. The STZ/HFD treatment decreased HOMA-β, but the effect was attenuated by TGPE (Figure 4A). Moreover, β-cell mass declined in the DM group (Figure 4B). Compared to the DM group, the 1X and 5X groups had higher β-cell mass. Pictures of the pancreas also showed that there were fewer β-cells in the STZ/HFD-treated groups, especially in the DM group (Figure 4C–F).

### 3.5. Serum Lipid Biochemical Parameters

The DM group showed higher levels of TC, TG, and LDL compared to the C and 5X groups. The DM group also had the lowest HDL. The 5X group had similar concentrations of TC, TG, HDL, and LDL to those of the C group (Table 2).

### 3.6. Pro-Inflammatory Cytokines and Antioxidant Factors

**Figure 5:**
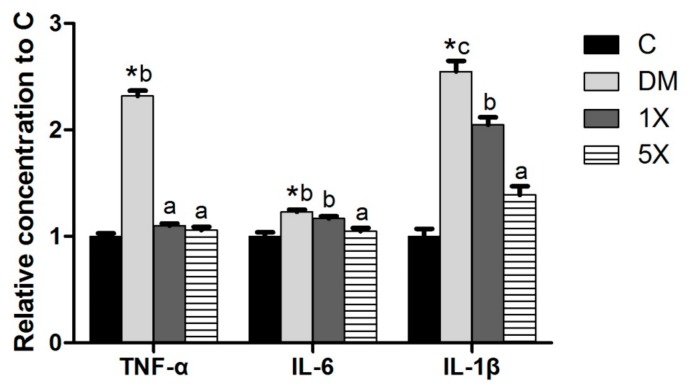
Serum inflammatory cytokines. C: vehicle control; DM: STZ/HFD vehicle control; 1X: STZ/HFD with 183.9 mg/kg/day of TGPE; 5X: STZ/HFD with 919.5 mg/kg/day of TGPE. Data are the means ± SEM (*n* = 10 rats/group). * Significantly different from the C group at *p* < 0.05 according to a one-tailed student’s *t*-test. Different superscript letters (a, b, c) indicate a significant difference at *p* < 0.05 according to one-way ANOVA with a Duncan post hoc test.

**Figure 6:**
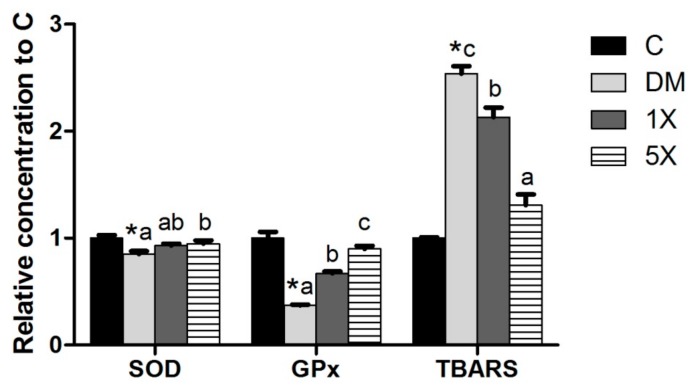
Serum antioxidant factors and TBARS. C, vehicle control; DM, STZ/HFD vehicle control; 1X, STZ/HFD with 183.9 mg/kg/day of TGPE; 5X, STZ/HFD with 919.5 mg/kg/day of TGPE. Data are the means ± SEM (*n* = 10 rats/group). * Significantly different from the C group at *p* < 0.05 according to a one-tailed student’s *t*-test. Different superscript letters (a, b, c) indicate a significant difference at *p* < 0.05 according to one-way ANOVA with a Duncan post hoc test.

Since inflammation and ROS are correlated with insulin resistance [22,23], pro-inflammatory cytokines and antioxidant factors were measured. To evaluate inflammation, we measured the levels of three pro-inflammatory cytokines: TNF-α, IL-6, and IL-1β. TNF-α, IL-6, and IL-1β were induced in the DM group, which was modulated in the 5X groups. The 1X group also showed lower serum concentrations of TNF-α and IL-1β compared to the DM group but had higher IL-6 and IL-1β serum concentrations than the 5X group (Figure 5). The antioxidant enzymes, SOD, and GPx decreased in the DM group, while their concentrations were close between the C and 5X groups. The TBARS assay, a well-established method for screening and monitoring lipid peroxidation, revealed the highest ROS in the DM group, followed by the 1X, 5X, and C groups (Figure 6).

### 3.7. Leptin and Adiponectin

**Figure 7:**
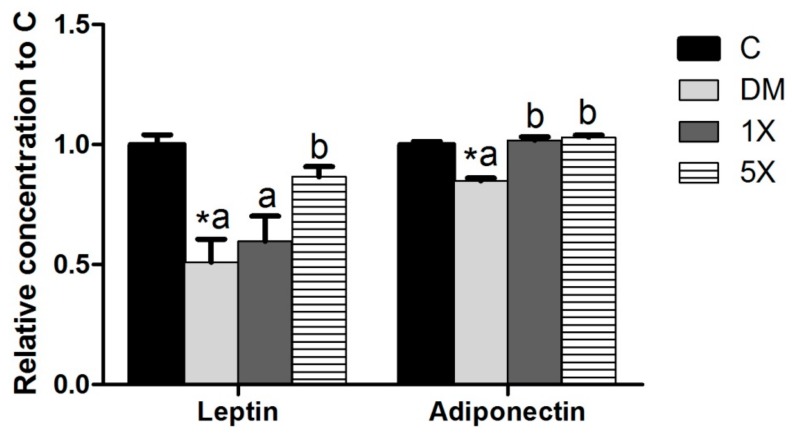
Serum leptin and adiponectin. C: vehicle control; DM: STZ/HFD vehicle control; 1X: STZ/HFD with 183.9 mg/kg/day of TGPE; 5X: STZ/HFD with 919.5 mg/kg/day of TGPE. Data are the mean ± SEM (*n* = 10 rats/group). * Significantly different from the C group at *p* < 0.05 according to a one-tailed student’s *t*-test. Different superscript letters (a, b) indicate a significant difference at *p* < 0.05 according to one-way ANOVA with a Duncan post hoc test.

The serum concentrations of leptin and adiponectin were also measured to study TGPE’s mechanisms of delaying diabetes. Both leptin and adiponectin were downregulated in the DM group. There were significant differences between the DM and 5X groups for leptin and adiponectin, while only adiponectin was upregulated in the 1X group (Figure 7).

### 3.8. mRNA Expressions of Lipid Metabolism Genes in the Liver

**Figure 8:**
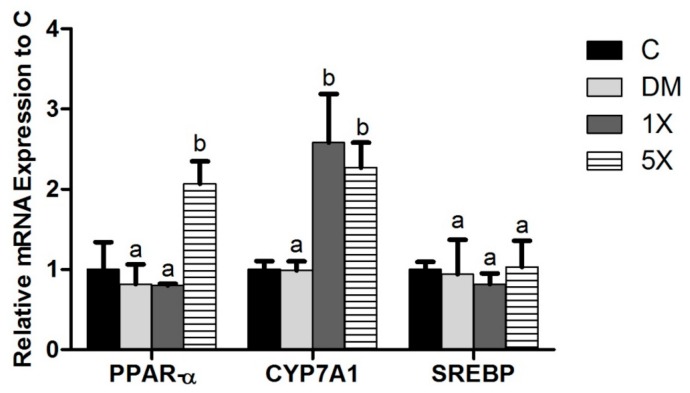
Relative expressions of hepatic lipid metabolism-related genes compared to the C group. C: vehicle control; DM: STZ/HFD vehicle control; 1X: STZ/HFD with 183.9 mg/kg/day of TGPE; 5X: STZ/HFD with 919.5 mg/kg/day of TGPE. Data are the means ± SEM (*n* = 10 rats/group). Different superscript letters (a, b) indicate a significant difference at *p* < 0.05 according to one-way ANOVA with a Duncan post hoc test.

Imbalanced lipid metabolism usually leads to insulin resistance. Due to the important role of the liver in lipid metabolism, we analyzed three hepatic lipid metabolism genes; *PPAR-α*, *CYP7A1*, and *SREBP*. *PPAR-α* was induced in the 5X group, and *CYP7A1* was upregulated in the 1X and 5X groups. The levels of the lipogenesis gene *SREBP* were not significantly different between all groups (Figure 8).

## 4. Discussion

In the present study, we demonstrated TGPE’s effect of delaying T2DM and highlight a potential underlying mechanism involving the attenuation of insulin resistance, protecting β cells, reducing oxidant and inflammatory factors, and inducing hepatic lipid catabolism and removal. Five dominant compounds were identified in the TGPE using HPLC: propolins C, D, F, G, and H. Propolins are isolated from the propolis in Taiwan and Japan and are different from the compounds from the propolis in other locations, such as Brazil and Europe [24].

Propolins C and D are prenylflavanone compounds nymphaeol-A and nymphaeol-B, respectively [25]. Propolin F is a prenylated flavonoid compound, isonymphaeol-B, and is isolated from Japanese propolis [26]. Propolins G and H are also prenylflavanones that can be isolated from Taiwanese green propolis and Taiwanese propolis [27,28]. Propolins C, D, F, and G have been reported to have anti-radical capabilities [4,26,27]. Free-radical scavenging activity is correlated with the improvement of diabetes, so it is not surprising to observe that T2DM was modulated by TGPE containing propolins C, D, F, G, and H in STZ/HFD-induced T2DM rats.

We also showed that the T2DM-delaying effect of TGPE occurred by delaying its progression. However, we did not examine which compound plays the most important role in this effect. Therefore, it would be interesting to investigate the individual propolin compounds separately from TGPE in the future.

The STZ/HFD rat model used in this study is modified from previous studies that started to treat the rats at the first week for a few weeks with multiple low doses of STZ and HFD to mimic the pathology of T2DM development [19,29]. The progression of T2DM begins with hyperglycemia, hyperinsulinemia, and insulin resistance, followed by β-cell failure in the final stage [13,30]. In our STZ/HFD animal model, the high FBG, FBI, and HOMA-IR in the DM group at week 5 (Figure 2 and Table 2) indicated that the DM group had hyperglycemia, hyperinsulinemia, and insulin resistance and was in the early stage of T2DM. At week 6, the sudden drop in FBI level and the high FBG level remaining in the DM group revealed the group had severe β-cell failure and was in the late stage of T2DM. Thus, the STZ/HFD treatment successfully mimicked the progression of T2DM in the rats.

Although the TGPE-treated groups showed hyperglycemia, hyperinsulinemia, insulin selectivity, and insulin resistance, severe β-cell failure was not observed in the 1X and 5X groups. Therefore, our results reasonably demonstrate that TGPE can extend the early stage of T2DM and delay into the late stage of T2DM. However, the experimental period was only eight weeks, so further study is needed to investigate how long the progression of T2DM can be delayed by TGPE.

Ninety per cent of diabetes cases are T2DM, which is caused by several factors, such as ROS, inflammation, lipid metabolism imbalance, and obesity [31]. Therefore, some functional foods that could decrease these factors were also reported to attenuate T2DM [32,33,34,35]. Although TGPE has not revealed an effect of diabetes modulation, its propolin components were found to increase the antioxidant cytokines SOD and GPx and decrease ROS [9,36]. Su et al. also showed that administering TGPE at 200 and 400 mg/kg/day significantly increased the secretion of GPx and SOD in mice in a dose-dependent manner [9].

According to the conversion constant between mice and rats from the FDA, the doses used by Su et al. in mice was equal to 101 and 202 mg/kg/day in rats [9]. Thus, the inductions of GPx and SOD by TGPE were unsurprisingly observed in the 1X group (183.9 mg/kg/day). For further testing of the dose-dependent effects of TGPE, we also treated the rats with a 5X dose of TGPE in this study. Our results revealed that the levels of GPx and SOD were higher in the 5X group than in the 1X group. Due to the negative correlation between the levels of TBARS and antioxidant enzymes, the results indicated that TGPE could reduce ROS by increasing the levels of GPx and SOD. Therefore, antioxidation might be the mechanism of TGPE in delaying the progression of T2DM.

Inflammation is an important factor that leads to insulin resistance and β-cell failure and speeds up the progression of T2DM [37]. Although different kinds of propolis are reported to have anti-inflammation abilities, the anti-inflammation effect of TGPE has not been shown in previous studies. For the first time, we presented that TGPE significantly decreases the inflammatory cytokines TNF-α, IL-6, and IL-1β in STZ/HFD-induced T2DM rats. Furthermore, TGPE could slow down the progression of T2DM. Thus, the oral administration of TGPE may also delay the progress of T2DM via anti-inflammation effects.

Improving lipid metabolism and obesity are also important for delaying the progression of T2DM [38]. The liver is a critical organ involved in the metabolism of lipids, carbohydrates, and proteins. Therefore, hepatic gene expressions related to lipid catabolism, bile acid production, and lipogenesis should affect T2DM. There are several important genes of lipid metabolism in the liver, such as *PPAR-α*, *CYP7A1*, and *SREBP*. *PPAR-α* is a major regulator of lipid metabolism in the liver, where it enhances the uptake and catabolism of fatty acids [39]. Since free fatty acids enhance insulin resistance and T2DM [38,40], increasing *PPAR-α* expression could decrease blood fatty acid and decrease ROS. Thus, it is not surprising that *PPAR-α* agonists are suggested as potential anti-T2DM drugs [41].

*CYP7A1* is another gene that assists in lipid removal and can be promoted by *PPAR-α* [42]. *CYP7A1* enhances the conversion of cholesterol to bile acid in the liver and increases the excretion of bile acid into the digestive tract [16]. *SREBP* is involved in fatty acid synthesis in the liver. *PPAR-α* and *CYP7A1* were suggested to be induced by bio- flavanone and flavonoid in liver cells [43,44,45]. Since TGPE contained bioflavanone (propolin C, D, G, and H) and bioflavonoid (propolin F), it was reasonable to observed the increases of hepatic *PPAR*-*α* and *CYP7A1* in the TGPE-treated groups. We have also demonstrated that TGPE-induced anti-hyperlipidemia is associated with the inductions of hepatic *PPAR-α* and *CYP7A1*, but not with the promotions of *SREBP*. Thus, the extra lipid from the HFD may be absorbed into the liver and then removed. TGPE may therefore attenuate the development and progression of T2DM by removing the extra lipids through *PPAR-α* and *CYP7A1*.

Koya-Miyata et al. revealed that Brazilian propolis also prevented HFD-induced hyperlipidemia, but they showed that Brazilian propolis reduced hepatic *SREBP*, and no differences were observed in hepatic *PPAR-α* and *CYP7A1* [46]. We further compared the hyperglycemia effects of TGPE and Brazilian propolis extract in T2DM rats. The results showed that treatments with TGPE (8 weeks, 183.9 mg/kg/day) and Brazilian propolis extract (10 weeks, 200 mg/kg/day) improved FBG levels by 48% and 19%, respectively [29]. Therefore, TGPE has better effects on lowering FBG than Brazilian propolis extract and appears to regulate hepatic lipid metabolism via a different pathway from Brazilian propolis.

Adipokines also play an important role against diabetes. Adiponectin is an adipokine that is correlated with insulin sensitivity. Leptin can correct either T1DM or T2DM in animal models. Clinical trials indicate that leptin increased insulin sensitivity in non-obese patients [47]. Therefore, the higher serum concentrations of adiponectin and leptin in the 1X and 5X groups in the present study indicate that TGPE may improve STZ/HFD-induced diabetes by stimulating the secretion of adiponectin and leptin in rats.

Dose is a considerable issue for the effects of functional food [48]. The dose we used in the 1X group was between the high and low doses in the study by Su et al., who also showed an increase of GPx and SOD [9]. To investigate the dose-dependent effects of TGPE on delaying T2DM progression, we also gave rats a 5X dose of TGPE. According to the results, the effect of delaying T2DM progression was better in the 5X group than the 1X group. Furthermore, the BW, food and water intakes, and serum biochemical parameters were between those of the C and DM groups. Moreover, the liver condition, activity levels, and exterior experience of the rats (data not shown) were not abnormal. Thus, our results indicated that both 183.9 and 919.5 mg/kg/day could safely induce the delaying effect on T2DM in STZ/HFD-treated rats.

Our results indicate that the TGPE has different molecular compounds and different action mechanisms in its anti-diabetes effects compared to Brazilian propolis. We also provided the first evidence demonstrating that the long-term oral administration of TGPE could successfully delay the development and progression of T2DM by reducing ROS and inflammation, balancing lipid metabolism in the liver, and increasing serum levels of adiponectin and leptin in STZ/HFD-induced T2DM rats. Since the effects of TGPE were involved in preventing but not curing T2DM, TGPE may be considered as a routine functional food supplement for delaying T237DM development in the future.

## Figures and Tables

**Table 1:** The effects of Taiwanese green propolis extract on BW, food intake, food consumption efficiency, and water intake in STZ/HFD-treated rats.

Parameter	C	DM	1X	5X
BW gain (g)/rat	195.5 ± 12.8	174.0 ± 12.1 *^,a^	190.4 ± 11.6 ^b^	196.6 ± 8.5 ^b^
Food intake (Kcal)/day/rat	98.3 ± 1.6	119.4 ± 1.8 *^,a^	118.9 ± 1.8 ^a^	118.9 ± 1.7 ^a^
FCE (%)	3.16 ± 0.06	2.22 ± 0.04 *^,a^	2.45 ± 0.05 ^b^	2.53 ± 0.03 ^b^
Water intake w0	34.8 ± 1.1	32.8 ± 0.8 ^a^	33.6 ± 1.0 ^a^	34.2 ± 1.0 ^a^
Water intake w8	34.3 ± 0.7	146.2 ± 4.3 *^,c^	104.3 ± 3.1 ^b^	65.4 ± 2.7 ^a^

C, vehicle control; DM, STZ/HFD control; 1X, STZ/HFD with 183.9 mg/kg/day of Taiwanese green propolis extract; 5X, STZ/HFD with 919.5 mg/kg/day of Taiwanese green propolis extract; FCE, food conversion efficiency (Increase of BW/total calorie intake). All values are means ± SEM (*n* = 10 rats/group). * Significantly different from the C group at *p* < 0.05 by one tail Student’s *t* test. Different superscript letters (a, b, c) indicate significant differences at *p* < 0.05 according to one-way ANOVA with a Duncan post hoc test.

**Table 2:** The effects of Taiwanese green propolis extract on serum biochemical profiles at the eighth week and ISI and HOMA-IR at the fifth week in STZ/HFD-treated rats.

Parameter	C	DM	1X	5X
FBG (mg/dL)	89.2 ± 1.5	415.3 ± 50.3 *^,c^	214.2 ± 19.0 ^b^	144.2 ± 8.1 ^a^
FBI (μg/L)	1.02 ± 0.01	0.41 ± 0.11 *^,a^	1.53 ± 0.22 ^b^	1.52 ± 0.09 ^b^
HbA1c (%)	4.33 ± 0.07	8.51 ± 0.27 *^,c^	6.91 ± 0.44 ^b^	5.49 ± 0.26 ^a^
TC	62.4 ± 2.07	85.4 ± 3.91 *^,b^	75.7 ± 3.90 ^b^	64.0 ± 4.43 ^a^
TG	46.0 ± 2.36	132.1 ± 3.06 *^,c^	72.9 ± 3.19 ^b^	51.2 ± 0.69 ^a^
HDL	28.0 ± 0.34	15.3 ± 0.14 *^,a^	17.2 ± 0.20 ^b^	22.2 ± 0.39 ^c^
LDL	4.1 ± 0.15	8.4 ± 0.16 *^,c^	7.8 ± 0.30 ^b^	5.3 ± 0.19 ^a^
ISI W5	−4.4 ± 0.1	−6.4 ± 0.2 *^,a^	−5.9 ± 0.2 ^b^	−5.4 ± 0.1 ^c^
HOMA-IR W5	5.05 ± 0.28	37.59 ± 7.18 *^,c^	21.66 ± 5.18 ^b^	13.36 ± 1.84 ^a^

C, vehicle control; DM, STZ + high-fat diet control; 1X, STZ + HFD with 183.9 mg/kg/day of Taiwanese green propolis extract; 5X, HFD with 919.5 mg/kg/day of Taiwanese green propolis extract. FBG, fast blood glucose; FBI, fast blood insulin; HbA1c, hemoglobin A1c; TC, total cholesterol; TG, triacylglycerol; HDL, high-density lipoprotein; LDL, low-density lipoprotein; ISI W5, insulin sensitivity indices at the fifth week; HOMA-IR W5, homeostatic model assessment of insulin resistance at the fifth week. All values are means ± SEM (*n* = 10 rats/group). * Significantly different from the C group at *p* < 0.05 according to one-tailed student’s *t*-test. Different superscript letters (a, b, c) indicate significant differences at *p* < 0.05 according to one-way ANOVA with a Duncan post hoc test.

## Supplementary Materials

The following are available online at http://www.mdpi.com/2072-6643/10/4/503/s1, Figure S1: Trends of body weight, Table S1: Ingredient composition of the diets fed to rats, Table S2: Primer sequences used in RT-qPCR.
