# Supplementary material for: Taiwanese Green Propolis Ethanol Extract Delays the Progression of Type 2 Diabetes Mellitus in Rats Treated with Streptozotocin/High-Fat Diet
Source: Nutrients. 2018 Apr 18;10(4):503. doi: 10.3390/nu10040503 (PMC5946288; doi:10.3390/nu10040503)
Supplement: Supplementary file 1 [file nutrients-10-00503-s001.zip › Supplemental table 1.docx]

| Supplemental table 1. Ingredient composition of the diets fed to rats | | |
| --- | --- | --- |
| **Ingredient composition** | **Normal diet** | **High-energy diet** |
| Corn starch | 46.23% | 4.50% |
| Dextrin | 15.38% | 1.49% |
| Casein-vitamin free | 13.89% | 13.96% |
| Sucrose | 9.92% | 27.89% |
| Fructose | 0.00% | 19.91% |
| Powdered cellulose | 4.96% | 4.70% |
| Soybean Oil | 3.97% | 21.90% |
| AIN 93M Mineral Mix | 3.47% | 3.47% |
| AIN 93 Vitamin Mix | 0.99% | 0.99% |
| Choline bitartrate | 0.23% | 0.23% |
| L-Cystine | 0.17% | 0.17% |
| t-Butylhydroquinone | 0.79% | 0.79% |
| kcal/g | 3.8 | 4.50 |
