# Supplementary material for: Taiwanese Green Propolis Ethanol Extract Delays the Progression of Type 2 Diabetes Mellitus in Rats Treated with Streptozotocin/High-Fat Diet
Source: Nutrients. 2018 Apr 18;10(4):503. doi: 10.3390/nu10040503 (PMC5946288; doi:10.3390/nu10040503)
Supplement: Supplementary file 1 [file nutrients-10-00503-s001.zip › Supplemental Table 2.docx]

| Supplemental table 2. Primer sequences used in RT-qPCR | | | |
| --- | --- | --- | --- |
| **Genes** | **Gene accession numbers** | **Forward primers** | **Reverse primers** |
| *PPAR-α* | NM_013196.1 | CCTTACCCTTGGAGATGAAG | CAGTAGCAGGAAGTCTTAGAAG |
| *CYP7A1* | NM_012942.2 | TGCCCATTTCTCATACACAGA | GTGCCTTCCCTCCAAGTATT |
| *SREBP-1* | NM_001276708.1 | CGACTACATCCGCTTCTTACAG | AAGCTGACACCAGGTCTTTC |
| *β-ACTIN* | NM_031144.3 | ACAGGATGCAGAAGGAGATTAC | ACAGTGAGGCCAGGATAGA |
