# Supplementary figures and images for: Taiwanese Green Propolis Ethanol Extract Delays the Progression of Type 2 Diabetes Mellitus in Rats Treated with Streptozotocin/High-Fat Diet
Source: Nutrients. 2018 Apr 18;10(4):503. doi: 10.3390/nu10040503 (PMC5946288; doi:10.3390/nu10040503)

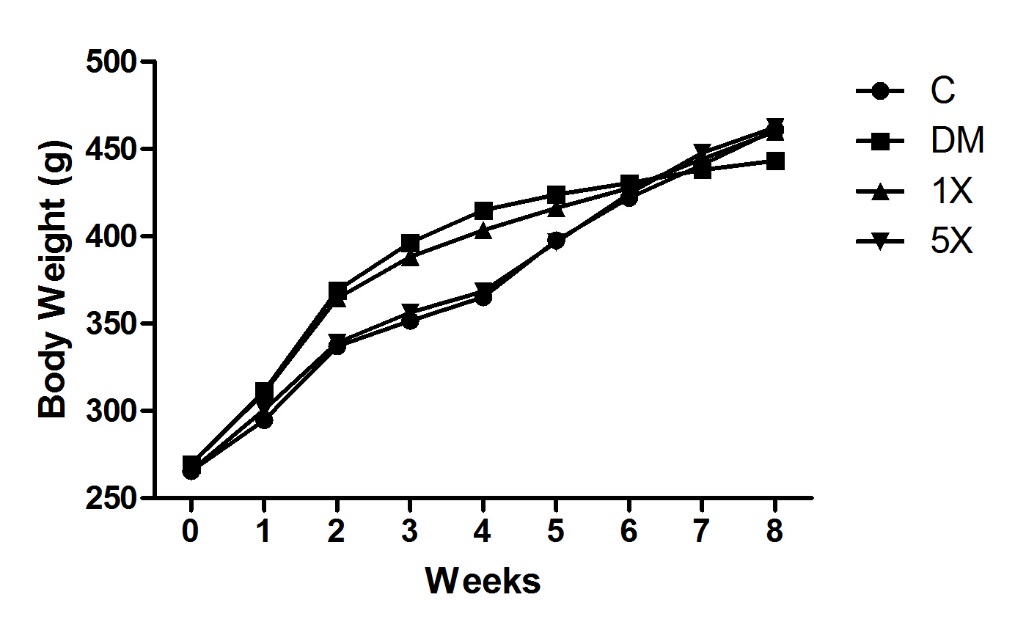


Figure 1. Trends of body weight

Supplement: Supplementary file 1 [file nutrients-10-00503-s001.zip › Supplemental figure 1.docx]
